# MicroRNAs as Indicators into the Causes and Consequences of Whole-Genome Duplication Events

**DOI:** 10.1093/molbev/msab344

**Published:** 2021-12-03

**Authors:** Kevin J Peterson, Alan Beavan, Peter J Chabot, Mark A McPeek, Davide Pisani, Bastian Fromm, Oleg Simakov

**Affiliations:** 1 Department of Biological Sciences, Dartmouth College, Hanover, NH, USA; 2 School of Earth Sciences, University of Bristol, Bristol, United Kingdom; 3 School of Biological Sciences, University of Bristol, Bristol, United Kingdom; 4 Arctic University Museum of Norway, UiT, The Arctic University of Norway, Tromsø, Norway; 5 Department of Neuroscience and Developmental Biology, University of Vienna, Vienna, Austria

**Keywords:** microRNA, whole-genome duplication, complexity

## Abstract

Whole-genome duplications (WGDs) have long been considered the causal mechanism underlying dramatic increases to morphological complexity due to the neo-functionalization of paralogs generated during these events. Nonetheless, an alternative hypothesis suggests that behind the retention of most paralogs is not neo-functionalization, but instead the degree of the inter-connectivity of the intended gene product, as well as the mode of the WGD itself. Here, we explore both the causes and consequences of WGD by examining the distribution, expression, and molecular evolution of microRNAs (miRNAs) in both gnathostome vertebrates as well as chelicerate arthropods. We find that although the number of miRNA paralogs tracks the number of WGDs experienced within the lineage, few of these paralogs experienced changes to the seed sequence, and thus are functionally equivalent relative to their mRNA targets. Nonetheless, in gnathostomes, although the retention of paralogs following the 1R autotetraploidization event is similar across the two subgenomes, the paralogs generated by the gnathostome 2R allotetraploidization event are retained in higher numbers on one subgenome relative to the second, with the miRNAs found on the preferred subgenome showing both higher expression of mature miRNA transcripts and slower molecular evolution of the precursor miRNA sequences. Importantly, WGDs do not result in the creation of miRNA novelty, nor do WGDs correlate to increases in complexity. Instead, it is the number of miRNA seed sequences in the genome itself that not only better correlate to instances in complexification, but also mechanistically explain why complexity increases when new miRNA families are established.

## Introduction

The origins of vertebrate complexity relative to most invertebrate taxa have long been sought in whole-genome duplication (WGD) events (Ohno 1970). Various vertebrate lineages have experienced WGDs, with one (known as 1R) occurring after the divergence of the vertebrate lineage from invertebrates, but before the vertebrate last common ancestor (LCA), and a second (known as 2R) after the divergence of gnathostomes from cyclostomes, but before the gnathostome LCA (Lundin et al. 2003; Dehal and Boore 2005; Putnam et al. 2008; Simakov et al. 2020; Lamb 2021; Nakatani et al. 2021). Each of these two rounds of WGD would have doubled the genic content of the organism, and although most of these newly duplicated genes would be lost, some—through what Ohno (1970) called “forbidden mutations”—would be retained and now able to explore new evolutionary avenues normally not available to the gene product. Through this process of neofunctionalization, these genes would find new roles to play in vertebrate biology, and as Ohno (1970) first argued, would ultimately allow for an increase to their organismal complexity relative to most invertebrates (see also, Holland et al. 1994; Sidow 1996; Escriva et al. 2006; Freeling and Thomas 2006; Putnam et al. 2008; Van de Peer et al. 2009, 2017; Huminiecki and Heldin 2010; Cañestro et al. 2013; Glasauer and Neuhauss 2014; Yamada et al. 2021).

Nonetheless, as Ohno (1970) also recognized, an alternative explanation behind gene retention following WGDs could be for reasons that have nothing to do with genic novelty per se. The gene-dosage (or gene-balance) model of selective gene retention (Veitia 2002; Papp et al. 2003; Birchler et al. 2005; Freeling and Thomas 2006) posits that genes whose products interact with other gene products in precisely determined stoichiometric ratios—in particular genes that encode for transcription factors, components of signal transduction pathways, and cell-cycle proteins—are selectively retained following WGDs, in contrast to gene products that are not under similar constraints, and hence return to single copy genes following WGDs (see also, Blanc and Wolfe 2004; Seoighe and Gehring 2004; Birchler et al. 2005; Blomme et al. 2006; Conant and Wolfe 2008; Edger and Pires 2009; Hufton et al. 2009; Kassahn et al. 2009; Makino et al. 2009; Huminiecki and Heldin 2010; Makino and McLysaght 2010; Birchler and Veitia 2012; Buggs et al. 2012). Thus, the loss of newly generated paralogs from WGDs is not random with respect to the encoded gene product, but instead dependent upon its connectivity to other gene products (Gibson and Spring 1998; Veron et al. 2007). Consistent with this insight, dosage-sensitive genes—at least in human—are rarely found in tandem pairs, are often associated with haploinsufficiency, have significantly more protein interactions than the genomic mean, and are enriched in collections of disease-related genes relative to dosage-insensitive genes (Kondrashov and Koonin 2004; Birchler et al. 2005; Blomme et al. 2006; Makino and McLysaght 2010; Birchler and Veitia 2012; Singh et al. 2012).

Beyond the functional categorization of the gene product, a second reason why the loss of paralogs following WGDs is often not random involves the mode of the WGD event itself. There are two types of WGD (Ohno 1970; Garsmeur et al. 2014). The first—autopolyploidy—is when a mistake in DNA replication occurs relative to cytokinesis (Comai 2005) generating an entire second copy of the organism’s genome. Because of this identity, the subsequent elimination of these newly generated paralogs during the re-diploidization process is effectively random with respect to which of the two genomes housed the newly lost gene (Garsmeur et al. 2014). However, in instances of allopolyploidy in which two different diploid species hybridize, bringing together two distinct genomes into a single cell, the subsequent loss of paralogs—known as “homeologs” to distinguish them from the “ohnologs” generated during autotetraploidy (Glover et al. 2016)—is decidedly nonrandom. Instead, this rediploidization process results in “subgenome dominance” or “genome fractionation” where one of the two hybridized genomes is preferentially retained relative to the other (Thomas et al. 2006; Schnable et al. 2011; Garsmeur et al. 2014; Session et al. 2016; Cheng et al. 2018; Edger et al. 2018). Therefore, during instances of autotetraploidy, biases in gene retention will be seen with specific kinds of genes in terms of their encoded gene products, but in instances of allotetraploidy, biases in gene retention will be seen both with respect to the kind and the genomic location of the gene itself for reasons that have nothing to do with potential neo-functionalizations.

**Fig. 1.**
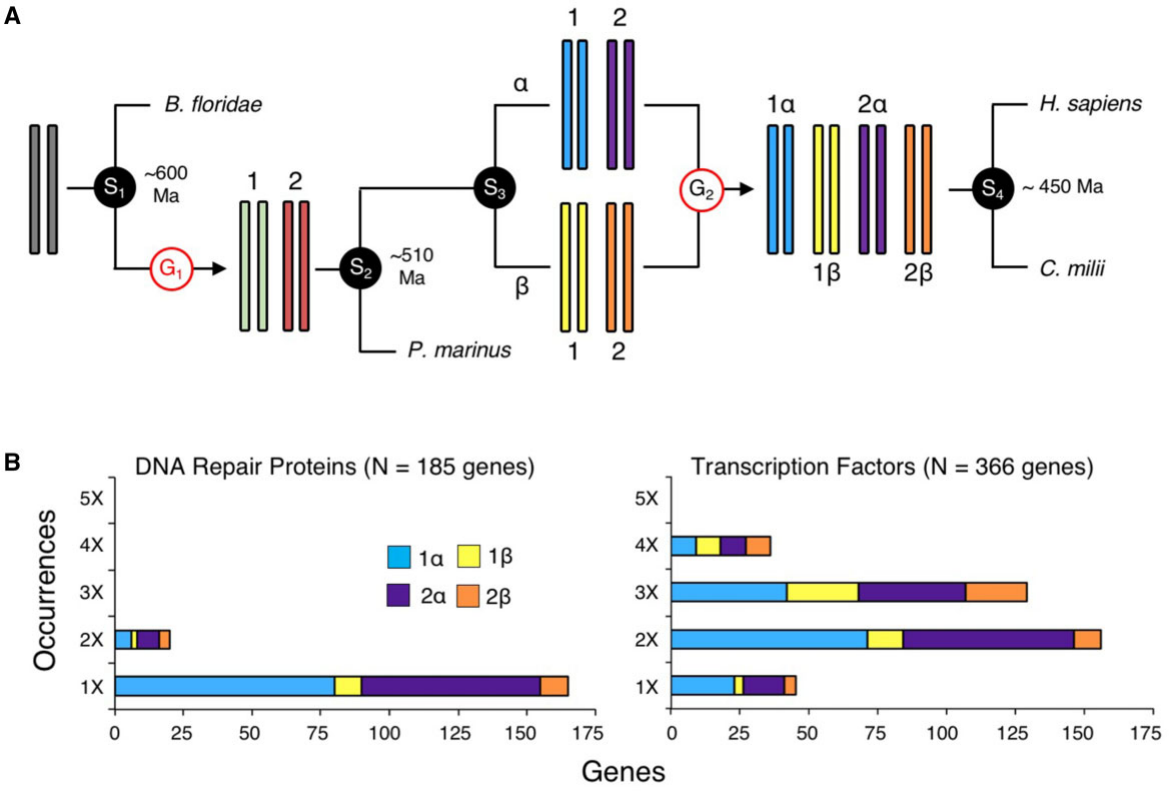
The Simakov et al. (2020) model of vertebrate genome evolution. (*A*) Starting from an initial diploid state of an early chordate ancestor, sometime after the split (speciation [S] event 1) from the invertebrate chordates (e.g., the amphioxus *Branchiostoma floridae*), but before the separation of the extant jawless fish (e.g., the lamprey *Petromyzon marinus*) from the jawed fish (S2), the genome doubled in content (G1) generating a tetraploid genome. Because the retention of genes does not differ between subgenomes 1 and 2, Simakov et al. (2020) reconstructed this WGD as an autotetraploidy event. Then, sometime after the vertebrate LCA (S2), there was a speciation event (S3) generating two, now extinct, lineages that Simakov et al. (2020) delineated α and β. After this speciation event, but before the gnathostome LCA (S4), there was a hybridization event between two species, one belonging to each of these two lineages, resulting in an allotetraploidization event (G2). Thus, the gnathostome genome—represented by *Homo sapiens* and the elephant shark *Callorhinchus milii*—is now octoploid with respect to the ancestral chordate genome. (*B*) Retention evidence for an auto- followed by an allotetraplodization event in the early vertebrate lineage. Shown are the genomic distributions of 175 genes that encode DNA repair proteins (left, updated from Wood et al. [2001]) and 175 genes that encode transcription factors (right, Lambert et al. 2018) that were present as single copy genes in the chordate LCA (supplementary tables 1 and 2 and file 1, Supplementary Material online). Each gene was placed on subgenome “1” or “2” and “α” or “β” following Simakov et al. (2020) and Lamb (2021), with each paralog in the genome separated by more than 50 kb from any other paralog. Importantly, even though transcription-factor encoding genes are maintained at a mean of 2× relative to the invertebrate amphioxus, whereas DNA-repair encoding genes are largely maintained as single copy, both show significant enrichment of genes on the α subgenome versus the β subgenome, but not between the 1 and 2 subgenomes (supplementary tables 1 and 2, Supplementary Material online).

Because of the nonrandomness of paralog losses from one of the two genomes following a hybridization event, allotetraploidy can be readily discerned from autotetraploidy simply by demonstrating the biased retention of genes from one subgenome relative to the other (Session et al. 2016). Simakov et al. (2020) explored retention rates of paralogs across select vertebrate genomes and discovered that 1R was an autotetraploidy event (fig. 1*A*), recognized by the parity of gene retention between subgenomes “1” and “2” for both dosage insensitive (e.g., DNA repair proteins, fig. 1*B*, left) as well as dosage sensitive (e.g., transcription factors, fig. 1*B*, right) gene products (see supplementary tables 1 and 2 and file 1, Supplementary Material online). However, 2R was an allotetraploidy event where two different species—termed α and β by Simakov et al. (2020)—hybridized (see also, Nakatani et al. 2021). Losses then preferentially accrued on the DNA derived from the β subgenome relative to the α subgenome, again for both dosage-insensitive and dosage-sensitive gene products (fig. 1*B*).

Why genes from one of the hybridized genomes is preferred over the other remains unknown. Several hypotheses have been proposed (reviewed in Edger et al. [2018]). One idea focuses on the hypothesis that the interactions between gene products governs retention such that only partners derived from the same genome would be retained (Thomas et al. 2006; Veitia 2010; Buggs et al. 2012). A second idea is that the differential expression of genes governs retention such that genes are retained from the genome that generated the higher transcript abundance due to potentially epigenetic differences between the two subgenomes (Gout et al. 2010; Session et al. 2016; Xu et al. 2019). We sought to discriminate between these two competing (but not necessarily mutually exclusive) hypotheses by examining the genomic distribution of microRNA (miRNA) genes across a representative sample of jawed vertebrates (gnathostomes), as well as other lineages that also experienced WGDs, in particular chelicerate arthropods. miRNAs encode ∼22 nucleotide noncodingRNA products that interact with target messenger RNAs (mRNAs) primarily through nucleotide positions 2–8 of the mature miRNA gene product, what is known as the “seed” (Bartel 2009, 2018; Dexheimer and Cochella 2020). This interaction between the seed sequence of a miRNA and a target mRNA results in the abrogation of the mRNA through the activity of the protein Argonaute that forms the enzymatic core of the RNAi-induced silencing complex (Schirle et al. 2014; Nakanishi 2016). Because the interaction between the miRNA seed sequence and the target mRNA sequence involves simple base-pairing rules between the two, the same seed sequence from different miRNA paralogs can potentially interact with the same set of mRNA targets. This then allows a test between these two hypotheses for subgenome dominance: if the selective retention of genes is primarily due to interactions between genic products—whether RNA or protein—this should result in randomness of miRNA retention between the α and β subgenomes of extant gnathostomes, given the strong conservation of the seed and 3′-CR regions of gnathostome miRNAs (Fromm et al. 2015). Alternatively, if the reasons for subgenome dominance center around the location of the gene itself, then miRNA paralog retention should follow the same trends that Simakov et al. (2020) demonstrated for protein-coding genes (fig. 1*B*).

Here, we show that similar to younger genome duplication events in fish (Berthelot et al. 2014) and *Xenopus* (Session et al. 2016) miRNAs follow the same retention trends as their principal targets and are selectively retained following WGDs. An examination of genomic retention unambiguously shows that gnathostome miRNAs—like their protein-coding genes—are selectively retained on the α genome relative to the β genome. However, unlike protein-coding genes (Simakov et al. 2020), miRNA paralogs are continually lost on the β subgenome relative to the α subgenome for hundreds of millions of years after the gnathostome LCA. Further, these β homeologs are expressed at lower levels, and experience more mutations to their mature sequence, than the homeologs found on the α subgenome. Finally, following Heimberg et al. (2008), we argue that WGDs are not primary drivers of morphological evolution. Instead, the best predictor of morphological and behavioral complexity in any animal lineage is the number of distinct miRNA seed sequences present in the genome itself, sequences that, surprisingly, are not the result of WGDs.

## Results

### Retention of miRNAs Following WGD Events

Aside from the studies of Bhambri et al. (2018) and Desvignes et al. (2021), most efforts to understand the increase in miRNA paralog numbers in metazoan taxa that have undergone WGD events (Hertel et al. 2006; Berthelot et al. 2014; Braasch et al. 2016; Leite et al. 2016; Shingate, Ravi, Prasad, Tay, Garg, et al. 2020; Nong et al. 2021) were hampered by the difficulty in assigning direct homology between individual miRNA genes. However, MirGeneDB (Fromm et al. 2015, 2020) was created with the specific intent to use a consistent nomenclature system that explicitly recognizes paralogs within a taxon and orthologs across taxa based on both syntenic and phylogenetic analyses. Therefore, the miRNA repertoire of any one species can be directly compared with any other within the database. In addition, the miRNA repertoire of extinct species can be easily reconstructed given that the evolutionary point of origin of every miRNA within the database (nearly 15,000 robustly identified miRNA loci from 73 metazoan taxa), including its family-level membership, is explicitly identified within the context of the database’s taxonomy.

To assess our methodology with respect to miRNA homology assignments, we constructed a concatenated data set of all 254 precursor miRNA sequences reconstructed as present in the gnathostome LCA (supplementary file 2, Supplementary Material online). Each of these 254 miRNA precursor sequences from 32 representative taxa was aligned, concatenated, and analyzed by Bayesian analysis (see Materials and Methods). Because the phylogeny of these 32 taxa is known, any deviation from this accepted topology could be due to one of several reasons including through mis-assignment of miRNA gene identities, or due to meiotic exchanges between homeologs that could have occurred after the hybridization event (Edger et al. 2018). However, we find robust support for this accepted topology with most nodes supported with high posterior probabilities (supplementary fig. 1, Supplementary Material online). The relatively low support of the eutherian nodes Glires and Atlanogenata is similarly difficult to capture with protein-coding genes (supplementary fig. 1, Supplementary Material online; see Materials and Methods), and thus appears to be clade-specific issues not related to difficulties with miRNA homology assignments.

**Fig. 2.**
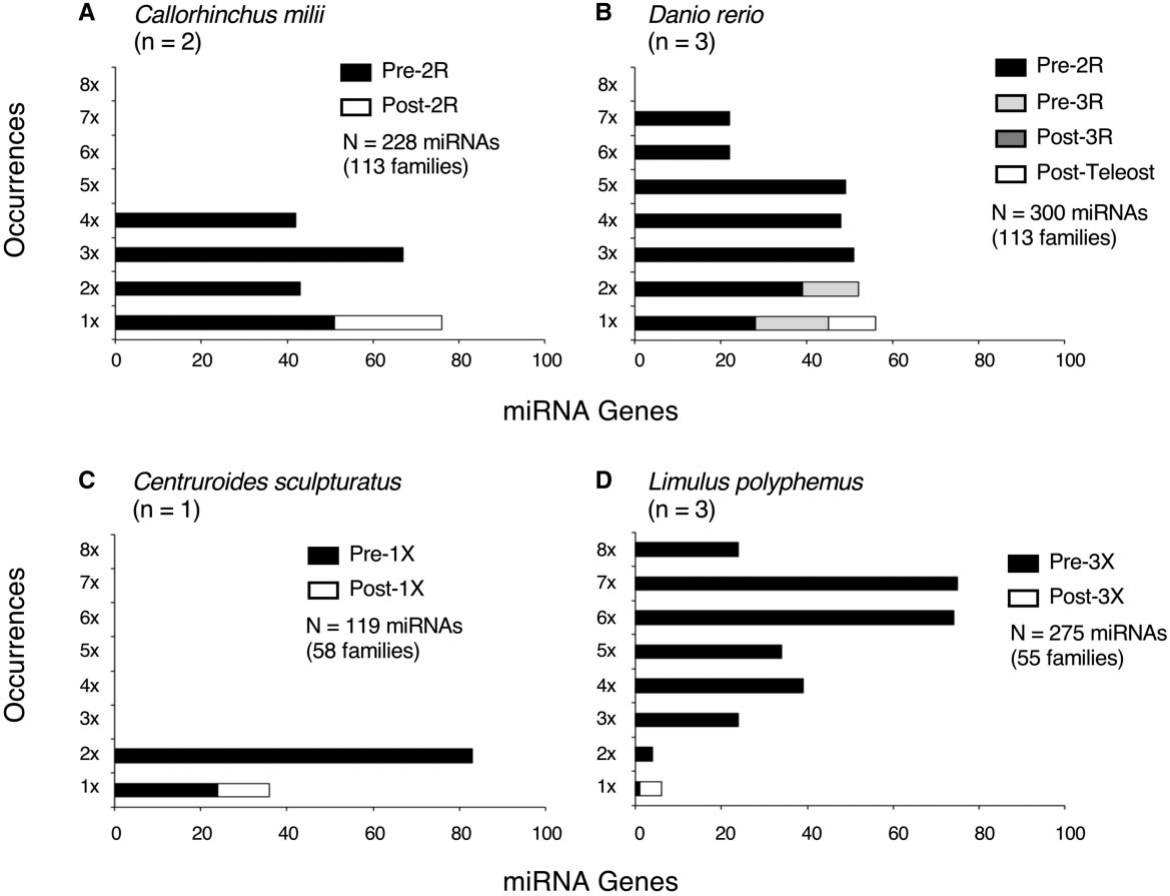
The occurrences of miRNA paralogs reflect the number of WGD events. Shown are the number of occurrences of miRNA paralogs in four different metazoan genomes, the gnathostomes *Callorhinchus milii* (elephant shark) (*A*) and *Danio rerio* (zebrafish) (*B*), and the chelicerates *Centruroides sculpturatus* (bark scorpion) (*C*) and *Limulus polyphemus* (Atlantic horseshoe crab) (*D*). In each case the maximal number of miRNA paralogs separated by at least 50 kb is simply equal (or nearly equal) to 2^*n*^, where *n* is the number of WGDs. Importantly, none of these WGDs resulted in a significant increase in the number of miRNA families, only paralogs to previously existing families.

Because our miRNA homology assignments appear robust, we next asked if the number of occurrences of miRNA paralogs corresponds to a taxon’s known number of genome duplication events (fig. 2). Profiling the distribution of miRNA paralogs within the genome of the shark *Callorhinchusmilii* taxa shows that it has numerous instances of up to four paralogs of miRNAs (but no more) distributed throughout the genome with no paralog separated by less than 50 kb from another, the distance used herein as the maximal extent of a miRNA polycistron (Baskerville and Bartel 2005). Interestingly, all of the miRNA paralogs in the gnathostome genome found at least twice belong to miRNA families that evolved before the LCA of all living vertebrates (fig. 2*A*, black bars), but none involving families that evolved after the vertebrate LCA (fig. 2*A*, white bar). The teleost fish *Danio rerio* though has up seven paralogs of miRNAs due to the 3R event that occurred in the teleost lineage after it split from the holostean fish like the gar, but before the teleost LCA (Glasauer and Neuhauss 2014; Desvignes et al. 2021). For miRNA families that evolved after 2R, but before 3R, these occur at no more than two times in the genome of *D. rerio* (fig. 2*B*, gray bars), and for those that evolved after 3R, these are found as genomic singletons (fig. 2*B*, white bar).

Similarly, within the chelicerates, we find that again miRNAs track the number of WGD events. Most arthropods including the tick *Ixodesscapularis* (Schwager et al. 2017; Shingate, Ravi, Prasad, Tay, and Venkatesh 2020) have not experienced any WGD, and thus have few if any miRNA paralogs separated by more than 50 kb from one another (MirGeneDB.org). However, the scorpion *Centruroides sculpturatus*, which has experienced a single WGD shared with spiders (Schwager et al. 2017), has numerous miRNA paralogs on separate contigs, but none on more than two (fig. 2*C*). Furthermore, the Atlantic horseshoe crab *Limulus polyphemus*, which like teleosts has undergone three WGDs (Nong et al. 2021), has miRNA paralogs occurring in the genome up to eight times for miRNA families that evolved before the WGD events (fig. 2*D*, black bars), but only singletons for miRNAs that evolved after the WGDs (fig. 2*D*, white bar). Therefore, similar to the genes that encode a subset of their principal targets (fig. 1*B*, right), miRNAs are retained as multiple paralogs following WGD events in both gnathostomes as well as in chelicerate arthropods, paralog numbers that reflect the number of WGDs themselves.

### The Distribution of miRNAs in the Genomes of Three LCAs

Because the gnathostome miRNAs are distributed throughout the genome in a manner that reflects the number of WGDs, we next sought to reconstruct the miRNA repertoire of three LCAs (Chordata, Vertebrata, and Gnathostomata). Simakov et al. (2020) confirmed that the chordate LCA had at least 17 linkage groups (Putnam et al. 2008; Sacerdot et al. 2018; Lamb 2021; Nakatani et al. 2021), and related these ancestral linkage groups (ALG) to the extant genomes of four key chordate taxa—the amphioxus *Branchiostomafloridae*, the chicken *Gallusgallus*, the spotted gar *Lepisosteusoculatus*, and the frog *Xenopustropicalis*. Thirty of 33 miRNA genes or gene clusters present in this LCA (MirGeneDB.org) could reliably be placed on one of these 17 ALGs (supplementary fig. 2*A*, Supplementary Material online). Twenty-six of these miRNAs or clusters of miRNAs would be passed on to the vertebrate LCA, and all but one (Mir-33) are still found on the same ancestral ALG (supplementary fig. 2*A* and *B*, Supplementary Material online, pound sign); an additional four miRNAs or clusters of miRNAs were lost after the chordate LCA, but before the vertebrate LCA (supplementary fig. 2*A*, Supplementary Material online, downward arrows).

The vertebrate LCA is reconstructed as having 34 linkage groups (supplementary fig. 2*B*, Supplementary Material online), a result of the first WGD event with no apparent chromosomal fusions or fissions (Simakov et al. 2020; Lamb 2021; Nakatani et al. 2021). The three unplaced miRNA families in the chordate LCA (MIR-34, MIR-92, and MIR-103) are all now placed on the genomic reconstruction of the vertebrate LCA. Of the 32 miRNAs or clusters of miRNAs present in the Olfactores LCA, 17 are now present in two copies, one on each of the two subgenomes, and 13 on either subgenome 1 or 2 (table 1). The vertebrate LCA has an additional 43 miRNA genes or clusters of genes (supplementary fig. 2*B*, Supplementary Material online, bold) that evolved after the Olfactores LCA, but before the vertebrate LCA. Nineteen of these are found on both subgenomes, and thus evolved before the autotetraploidy event. An additional 24 genes or clusters of genes are found on only one of the two subgenomes (supplementary fig. 2*B*, Supplementary Material online, asterisks), either because one ohnolog was lost, or because it evolved sometime between 1R and the vertebrate LCA. Comparisons with the prevertebrate miRNAs indicates that the former is more likely as there is no statistical difference between the retention of prevertebrate singletons versus vertebrate singletons (χ^2^ = 1.50, df* *=* *1, *P *=* *0.22). Therefore, although these miRNA distribution data are best explained by an autotetraploidy event, 1R did not result in the evolution of an unusually high number of novel miRNA families (Heimberg et al. 2008).

**Table 1.** miRNA Paralog Retention in Each of the Two Original Subgenomes (1 and/or 2) in the Vertebrate LCA.

Taxon	1 and 2	1	2
Prevertebrate^a^	16	7	7
Vertebrate^b^	19	9	15

a This row tabulates miRNA genes or clusters of genes that evolved before the Olfactores LCA and are found on both subgenomes 1 and 2, subgenome 1, or subgenome 2 in the vertebrate LCA (“Pre-V,” supplementary file 3, Supplementary Material online).

b This row tabulates miRNA genes or clusters of genes that evolved after the Olfactores LCA but before the vertebrate LCA and are found on both subgenomes 1 and 2, subgenome 1, or subgenome 2 in the vertebrate LCA (“V,” supplementary file 3, Supplementary Material online).

The genome of the gnathostome LCA is reconstructed as having at least 45 linkage groups, a result of the second tetraploidy accompanied with several chromosomal fusion events (supplementary fig. 2*C*, Supplementary Material online) (Simakov et al. 2020; Lamb 2021; Nakatani et al. 2021). Seventy-nine miRNA families were inherited from the vertebrate LCA, with paralogs distributed between one to four subgenomes (supplementary file 3, Supplementary Material online). An additional 11 families evolved after this second WGD, but before the gnathostome LCA (supplementary fig. 2*C*, Supplementary Material online, upward arrows), and are present as singletons in the genome of the gnathostome LCA. Thus, again consistent with Heimberg et al. (2008), 2R did not result in an influx of an unusually high number of new miRNA families into the gnathostome lineage (Fromm et al. 2015).

**Fig. 3.**
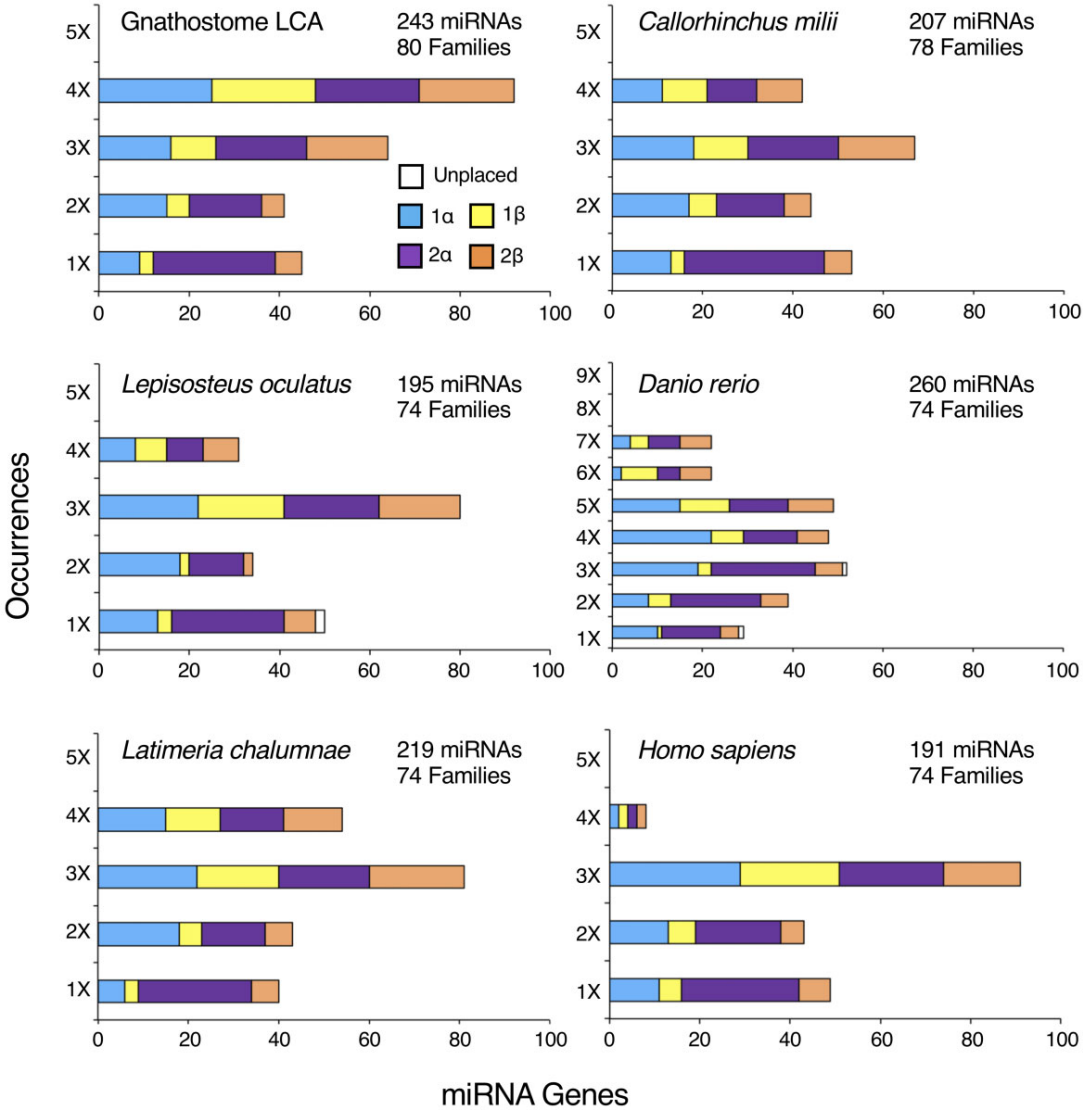
The distribution of miRNA paralogs across the four subgenomes in six representative gnathostome taxa. Tabulating the occurrences of miRNAs paralogs of miRNA families present in the vertebrate LCA shows that in each instance these paralogs are enriched on the α subgenome versus the β subgenome, but not between 1 versus 2 subgenomes (table 2). These observations are consistent with Simakov et al.’s (2020) hypothesis that the gnathostome 2R events were an allotetraploidization following an autotetraploidization (see fig. 1). Unexpectedly, in contrast to the protein-coding repertoire (Simakov et al. 2020), there is continued loss of β versus α paralogs long after the gnathostome LCA as seen in not only these five taxa, but in all extant gnathostome taxa analyzed (table 2). Both the number of paralogs and the number of families remaining in each extant taxon in relation to the gnathostome LCA are also indicated.

With this genomic reconstruction in hand, we can now ask if miRNAs show subgenome dominance as a result of the 2R event, but not due to the 1R event. Figure  3 shows the subgenome distributions of the miRNAs in the genome of the gnathostome LCA, as well as in the genomes of five select descendant taxa. In all cases, miRNAs are significantly enriched on the α subgenome relative to the β subgenome (χ^2^* *=* *95.6, df* *=* *1, *P *<* *0.0001), but are not enriched on subgenome 1 relative to subgenome 2. Thus, as demonstrated by Simakov et al. (2020) for protein-coding genes (fig. 1), miRNAs follow the same genomic biases that resulted from 2R allotetraploidy event. However, unlike protein-coding genes (Simakov et al. 2020), miRNA losses continue on the β subgenome relative to the α subgenome long after the gnathostome LCA (χ^2^* *=* *32.0, df* *=* *3, *P *<* *0.0001) (table 2; supplementary fig. 3 and file 3, Supplementary Material online). Thus, whatever the mechanism for biased gene retention following allotetraploidy events, this bias continues—at least for miRNAs—for hundreds of millions of years after the 2R event itself.

**Table 2.** miRNA Paralog Retention in Each of the Four Ancestral Subgenomes in the Gnathostome LCA and in Select Gnathostome Descendants.

	1α	2α	1β	2β
Total	65	86	41	50
4×^a^	25	23	23	21
3×^a^	16	20	10	18
2×^a^	15	16	5	5
1×^a^	9	27	3	6
Post-LCG^b^	54	73	64	93

a These rows tabulate the total number of miRNA genes present singularly or in clusters in four, three, two, or one copies in the genome of the gnathostome LCA (“LCG,” supplementary file 3, Supplementary Material online).

b This row tabulates miRNA losses specific to one of the four gnathostome subgenomes that occurred after the gnathostome LCA in 14 representative descendant taxa as detailed in supplementary figure 3 and file 4, Supplementary Material online.

### miRNA Sequence Expression and Evolution

**Fig. 4.**
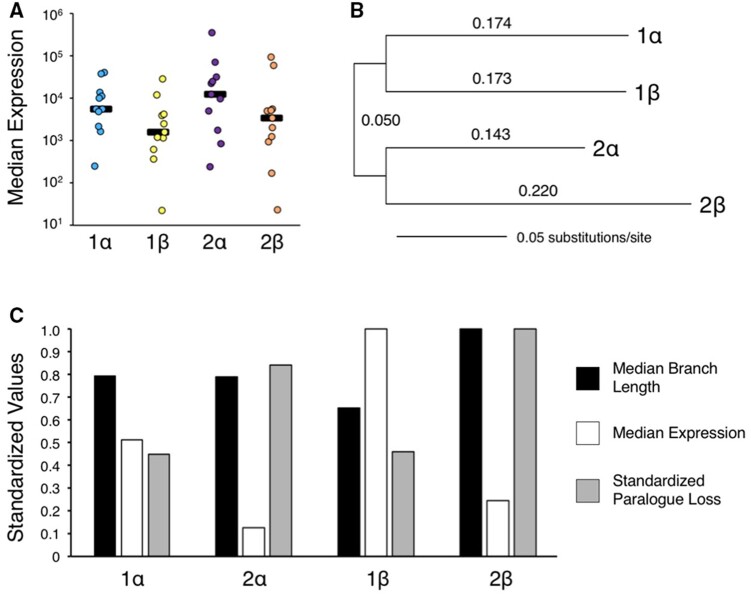
The expression and evolution of α versus β paralogs in extant gnathostomes. (*A*) Jigger plots of the median reads/million (rpm) values for unique miRNA mature sequences with at least one paralog on an α subgenome and one on a β subgenome in 11 extant gnathostome taxa. Note that mature miRNA expression is enhanced on α subgenomes versus β subgenomes (supplementary table 4 and file 5, Supplementary Material online). (*B*) Concatenating all paralogs present in 13 extant gnathostome taxa with at least one paralog on an α subgenome and one on a β subgenome (supplementary file 7, Supplementary Material online) shows that pre-miRNAs present on the 2α subgenome evolved significantly slower, and those present on the 2β subgenome significantly faster, than the paralogs present on the 1 subgenomes (supplementary table 4, Supplementary Material online). This mid-point rooted phylogram was constructed using maximum likelihood (see Materials and Methods); branch lengths are as indicated. (*C*) The inverse relationship between miRNA paralog retention and pre-miRNA molecular evolution versus miRNA expression present across the four subgenomes. The *y* axis indicates the standardized values to maximum for median expression (rpm, supplementary table 4, Supplementary Material online), median branch lengths (supplementary table 5, Supplementary Material online), and standardized paralog loss (table 2). miRNA loci found on the α subgenome are expressed significantly higher (supplementary table 3, Supplementary Material online) and retain significantly more miRNA loci (table 2; supplementary file 3, Supplementary Material online) than miRNA loci found on β subgenome. Furthermore, miRNAs found on the 2α subgenome evolved significantly slower than those on the 2β subgenome (supplementary table 4, Supplementary Material online).

Because there is a clear distinction between the retention of miRNA genes on the α versus β subgenomes, and the mature sequences for each set of paralogs is functionally equivalent—at least with respect to the sequence of the seed and most of the 3′-CRs (Fromm et al. 2015; supplementary fig. 5, Supplementary Material online)—we next asked if we could detect differences between either the expression of subgenome-specific sets of miRNAs, or the rate of primary sequence evolution of the pre-miRNAs themselves. We first compiled read data (standardized as reads per million) from MirGeneDB.org for 11 taxa where at least one α and one β subgenome houses a miRNA paralog generated by one or both of the vertebrate WGD events (supplementary file 5, Supplementary Material online). Importantly, only miRNAs with unique mature sequences were chosen for this analysis, greatly reducing the number of genes analyzed, but ensuring that the reads from multiple loci were not spuriously merged into one. Interestingly, the median expression from subgenome 1 is half that of subgenome 2, and from the β subgenome half that of the α subgenome (fig. 4*A* and *C*; supplementary table 3, Supplementary Material online) with the difference in median expression between α and β subgenomes significant (χ^2^* *=* *63,577.9, df* *=* *1, *P *<* *0.0001). Thus, as predicted from other independent allotetraploidy events (Session et al. 2016), the subgenome retaining the higher percentage of paralogs (in this case the α subgenome) also express miRNA genes at higher levels relative to the β subgenome. Unexpectedly though expression also shows a two-fold difference between the 1 versus 2 subgenomes.

We next asked if the rate of nucleotide substitution differs significantly across the four subgenomes generated from the two vertebrate WGD events (supplementary file 6, Supplementary Material online). Blanc and Wolfe (2004) showed that in *Arabidopsis* the subgenome with the higher percentage of gene retention exhibits a slower rate of molecular evolution in comparison to the second, gene-poor, subgenome, and we find the same to with vertebrates, at least with respect to the miRNAs found on the 2α subgenome relative to 2β (fig. 4*B*). We aligned the pre-miRNA sequences for each set of miRNA paralogs with at least one paralog on at least one of the two α subgenomes and a second on at least one of the two β subgenomes, and analyzed the concatenated sequences for each of the 13 taxa considered with maximum likelihood (ML, see Materials and Methods). This analysis shows that 2α, the subgenome most enriched for miRNA genes, evolves at a significantly slower rate than 2β, the subgenome most deficient in miRNA genes (*F*(1,48)* *=* *29.43, *P* < 0.0001) (supplementary table 4, Supplementary Material online). Taking the alignments for each individual taxon and concatenating them into a super-alignment shows a nearly identical set of branch lengths for each of the four subgenomes in comparison to the medians calculated from each taxon individually (fig. 4*B*;supplementary file 7, Supplementary Material online). Therefore, these data support a model that differences in gene expression, which in these two cases are correlated to differences in gene mutation, potentially lead to biases in genomic retention following allotetraploidy events.

## Discussion

The Simakov et al. (2020) model for the mode of the vertebrate WGD events was proposed given the disparity between gene retention on the α versus the β subgenome following 2R, but the parity of gene retention between the 1 and 2 subgenomes following 1R. Further, the timing of these two events was elucidated given that 2R is shared among all living gnathostomes, whereas 1R is shared with lampreys. Because MirGeneDB.org explicitly homologizes miRNAs within a taxon and between metazoan taxa, as well as identifies the node of origin of every miRNA locus as well as family, the mode and the timing of the 1R and 2R events can be assessed independently with a noncoding RNA marker. Here, we have shown that in both chelicerates as well as in gnathostome vertebrates, miRNAs are retained following WGD events in a manner reflecting the number of WGD events themselves (fig. 2). Further, within gnathostomes, miRNAs follow a similar pattern to the protein-coding repertoire (fig. 1) with miRNA homeologs enriched on the α subgenome relative to the β subgenome, but parity seen with ohnologs found on the subgenomes 1 versus subgenome 2 (fig. 3; supplementary fig. 2, Supplementary Material online; and table 1). Because the conservation of mature miRNA sequences among paralogs generated by either the 1R and/or 2R event(s) this biased retention of miRNAs is not due to target interactions with mRNA 3′-UTRs, but instead due to the genomic origin of the miRNA locus itself. Indeed, miRNA paralogs from the α subgenome show both higher expression of miRNA transcripts and—at least for 2α—slower molecular evolution of the precursor miRNA sequences relative to paralogs found on the β subgenome. Finally, none of the WGDs in either vertebrates or chelicerates resulted in the acquisition of an unusually high number of novel miRNA gene families. Instead, when dramatic increases to miRNA repertoires do occur, they are independent of WGD events, and it is these acquisitions of miRNA families—not WGDs—that are the likely reason behind increases in morphological and behavioral complexity in metazoans.

### Parity in miRNA Function but Nonparity in miRNA Retention, Expression, and Evolution

An outstanding question concerning WGDs is identifying the mechanism underlying subgenome dominance following allotetraploidy events. Several hypotheses have been advanced, principally involving either interactions of gene products or expression-level differences between the two newly hybridized genomes. Because homeologs were once orthologs that had independent evolutionary histories before the hybridization event, the coevolution of interacting gene products in one genome may occur in a different manner relative to the interacting gene products in the second. Thus, similar to a Bateson-Muller-Dobzhansky mechanism of incompatibility (Orr 1996), these two sets of gene products can only work with their partners from the same genome. Therefore, after the hybridization event, one set will be preferentially lost during the rediploidization process relative to the other, even for genes maintained in multiple copies for dosage reasons (fig. 1*B*, right). One might expect then that one set of interacting gene products would not necessarily reflect the genomic origin of another, independent set of interacting gene products. However, the fact that both DNA repair proteins and transcription factors all show α relative to β dominance (fig. 1*B*) suggests that a more likely reason behind subgenome dominance is the DNA itself, with one set of entire chromosomes preferred over the other. MicroRNAs offer an independent test of these ideas. One difficulty in understanding potential incompatibilities between two sets of gene products is simply understanding the detailed nature of the interactions themselves. However, miRNAs interact with messenger RNAs largely through their seven-nucleotide seed sequence (sometimes supplemented with the 3′-CR), and thus understanding the potential redundancy between homeologs is, at least in principle, far simpler with miRNAs than with protein sequences. With respect to the gnathostome WGD events, because there are no changes to the seed sequences after either WGD (supplementary fig. 5, Supplementary Material online), miRNAs from either the α versus the β genome should be interchangeable among themselves relative to the genomically preferred mRNA interactors(s). However, not only are miRNAs also strongly preferred on the α subgenome relative to the β, this preference continues into descendant taxa long after the allotetraploidy event itself (supplementary fig. 3, Supplementary Material online and table 2).

Consistent with this continual loss of miRNA paralogs on the β subgenome relative to the α is the fact that miRNA paralogs are expressed at higher levels on the two α subgenomes relative to the β subgenomes (fig. 4*A* and supplementary table 3, Supplementary Material online). Further, the 2α subgenome evolves much slower relative to the 2β subgenome (fig. 4*B*;supplementary table 4, Supplementary Material online), the subgenomes with the most and least miRNA paralogs, respectively (table 2). Indeed, there is a striking and statistically significant relationship between the subgenome placement of miRNAs generated during the 2R events, the expression levels of α versus β paralogs, and the branch lengths leading to the 2α versus the 2β paralogs in 13 representative gnathostome taxa (fig. 4*C*). Thus, the gnathostome genome is partitioned into four subgenomes, not only in terms of gene content (Simakov et al. 2020; Lamb 2021; Nakatani et al. 2021), but also in terms of miRNA gene expression and evolution. How these subgenomes maintain their identity for hundreds of millions of years after the 2R events themselves, and if these signals extend to other gene types beyond miRNAs, remain open questions.

### miRNAs, WGDs, and Phenotypic Complexity

WGD events have long enjoyed center stage as the mechanism for driving changes to phenotypic complexity (or species diversity when obvious changes to complexity are not apparent as in teleost fishes relative to other gnathostomes). As originally envisioned by Ohno (1970), because the ancestral vertebrate genome was duplicated in a single event, gene dosage was maintained where needed, with most other ohnologs lost through pseudogenization. However, some of these ohnologs hit upon mutations that gave them new roles to play in vertebrate construction and homeostasis, resulting in a dramatic increase to organismal complexity. Nonetheless, although elegant in its simplicity, this hypothesis is not supported by two observations. First, although bona fide instances of sub and neo-functionalization occurred after both the 2R event and the 3R event (Prince 2002; Escriva et al. 2006; Kenny et al. 2016; Yamada et al. 2021), the fact that ohno- and homeologs are enriched for gene products whose correct stoichiometric relationships with other gene products is essential, suggests that these instances of sub and neo-functionalization are likely exaptations (Gould and Vrba 1982), not adaptations, of the WGD events themselves (Freeling and Thomas 2006; Conant et al. 2014; Thompson et al. 2016; Clark and Donoghue 2018). For example, the instances of novelty—whether sub or neofunctionalization—documented in the *Hox* clusters of mammals and teleosts show that virtually all instances are specific for either the mammal or teleost lineage (Yamada et al. 2021). Thus, like the changes documented herein to mature miRNA sequences (supplementary fig. 4, Supplementary Material online), these are instances of exaptations that occurred long after the 1R and 2R events. Second, as argued by Donoghue and Purnell (2005), correlations between changes to phenotypic complexity (or diversity) and WGDs are only apparent when extant taxa are considered in isolation. When the fossil record is also considered, this apparent correlation breaks down as there is neither a burst of phenotypic innovation nor a change to diversity that could result from any of the WGDs known to have occurred in vertebrate evolution (see also Clarke et al. 2016; Davesne et al. 2021).

Similar to plants (Clark and Donoghue 2018), the purported relationship between WGDs and complexity is also now not supported by a broader appreciation of the frequency of WGDs throughout metazoans themselves. Indeed, the discovery of multiple WGDs in cyclostomes (Nakatani et al. 2021), in at least two chelicerate lineages (see fig. 2), as well as the oligochaete worm *Eisenia fetida* (Bhambri et al. 2018), the flatworm *Macrostomum lignano* (Wasik et al. 2015), and even in bdelloid rotifers (Mark Welch et al. 2008; Hur et al. 2009; Flot et al. 2013; Nowell et al. 2018), highlights that there is certainly no necessary correlation between WGD and increases to organismal complexity or species diversity, and certainly no “drive” (Freeling and Thomas 2006) toward increased morphological complexity on the heels of WGDs in either plants or animals. Indeed, as Kenny et al. (2016) emphasized, the fact that the classic living fossil—the horseshoe crab—consists of only four extant species and has shown little appreciable change in morphology since the Silurian (Briggs et al. 2012), despite undergoing three WGDs (fig. 2) sometime before the Early Cretaceous, highlights this absence of correlation, undermining any argument of necessary causality.

To address this disparity between the potential effects WGD had on vertebrate evolution versus chelicerate evolution, Kenny et al. (2016) suggested examining the patterns of miRNA innovation and loss following the WGDs in both. Here, we have revealed three very interesting patterns relevant to their suggestion. First, similar to their principal targets (fig. 1*B*, right), miRNA paralogs generated by WGDs are retained after these events in numbers that reflect the number of WGD events themselves (fig. 2). Nonetheless, this biased retention of miRNAs is not primarily due to neo-functionalizations either in gnathostomes (supplementary fig. 4, Supplementary Material online) or in the chelicerates (supplementary fig. 5, Supplementary Material online). Instead, as seen in plants (Abrouk et al. 2012), the retention of miRNA paralogs seems to be driven largely by gene dosage considerations between miRNAs and their target mRNAs, in particular transcription factors (fig. 1*B*, right). As several studies have demonstrated, the maintenance of the correct stoichiometry between miRNA mature molecules and the number of miRNA response elements in the 3′-UTRs of mRNAs (Denzler et al. 2014, 2016) is of considerable importance for the normal development and homeostasis of the cell (Broderick and Zamore 2014). Further, within gnathostomes, miRNA paralogs are rarely generated through tandem gene duplication, events with the potential to disrupt the stoichiometry between regulator and target. In fact, within the vertebrate set of paralogs generated through the 2R events, not a single shared tandem event occurred over the subsequent 450 My in any of our 14 representative gnathostomes lineages (supplementary fig. 4, Supplementary Material online). In fact, just under half (11 of 23 tandem pairs) of these potential, species-specific, gene duplicates have identical pre-miRNA sequences, consistent with the general observations of Rhie et al. (2021) that at least some are likely the result of mis-assembly of the genome itself. Therefore, despite a few potential examples of neo-functionalization (supplementary figs. 5 and 6, Supplementary Material online), these miRNA retention data suggest that very little novelty in terms of regulatory circuits arise following WGDs in either vertebrates or chelicerates.

The second striking pattern is that in all WGD cases examined herein, not once did a WGD event result in a demonstrable increase to the number of miRNA families, only paralogs to previously existing families (fig. 2) (Heimberg et al. 2008; Fromm et al. 2015). Even with the origin of the vertebrate-specific miRNA repertoire, whose acquisition occurred sometime around the first autotetraploidy event, nonetheless it is likely that most of these new miRNA families were already in place within the pre-1R genome. This is because miRNA families that were certainly in place before the 1R event are distributed in a statistically similar manner (table 1, χ^2^* *=* *1.50, df* *=* *1, *P *=* *0.22) to vertebrate-specific singletons that evolved after vertebrates split from urochordates (supplementary fig. 2, Supplementary Material online). This is a curious observation and raises the question of why WGDs do not generate an influx of new miRNA families given that not only is there a doubling of the number of introns—the predominant source of new miRNAs (Nozawa et al. 2010; Campo-Paysaa et al. 2011)—and a doubling of potential target sequences as well. Nonetheless, the absence of miRNA innovation following WGDs in both chelicerates and in vertebrates appears robust.

Instead—and this brings us to the final pattern—pulses of mRNA innovation occur for reasons other than WGDs, reasons that remain speculative at the moment. Nonetheless, it is these dramatic increases in the number of miRNA families and not WGDs that correlate to discrete advents of morphological complexity (Sempere et al. 2006; Heimberg et al. 2008; Peterson et al. 2009; Deline et al. 2018). Three large increases to the rate of miRNA innovation were known across the metazoan kingdom, and in each instance accompanied by an increase in cell-type complexity: at the base of bilaterians, at the base of vertebrates, and at the base of eutherian mammals (Hertel et al. 2006; Sempere et al. 2006; Prochnik et al. 2007; Heimberg et al. 2008; Wheeler et al. 2009; Tarver et al. 2013; Fromm et al. 2015). With each of these documented increased to the miRNA family-level repertoire, clade-specific miRNAs are often expressed in clade-specific tissues (Devor and Peek 2008; Christodoulou et al. 2010; Heimberg et al. 2010; Bartel 2018; DeVeale et al. 2021), suggesting that the former might be instrumental in the evolution of the latter (Sempere et al. 2006; Peterson et al. 2009). Indeed, with each novel seed sequence added to a genome, a new post-transcriptional regulatory circuit can now be established, bringing additional robustness to the developmental process (Heimberg et al. 2008; Li et al. 2009; Ebert and Sharp 2012; Cassidy et al. 2013; Schmiedel et al. 2015), increasing the heritability of the interaction (Hornstein and Shomron 2006; Peterson et al. 2009; Wu et al. 2009), and ultimately allowing for the evolution of new cell types and functions (Sempere et al. 2006; Deline et al. 2018).

## Materials and Methods

All miRNA data, including sequences, expression, and homology assignments, were taken from MirGeneDB.org (https://new.mirgenedb.org/). MirGeneDB identifiers for release 2.1 (Fromm et al. 2021) were updated from release 2.0 (Fromm et al. 2020) to reflect the subgenome locations of pre-1R miRNA families such that P1 = 1α, P2 = 2α, P3 = 1β, and P4 = 2β (supplementary file 3, Supplementary Material online) except that, as argued by Lamb (2021), the β1 versus β2 of linkage groups B, G, H were switched, as were the α1 versus α2 of linkage group A (paralogon 3 of Lamb 2021). Further, all miRNA paralog clusters included herein are now numbered so that -P1, for example, is 5′ of -P2, and that all linked genes are given the same linkage identifiers (e.g., Mir-1-P1 and Mir-133-P1 are clustered together, as are Mir-1-P2 and Mir-133-P1). See Fromm et al. (2021) for further details and examples. The vertebrate pre1-R set of miRNAs is taken from the shared complement of miRNA families present in both gnathostomes and cyclostomes as lamprey shares the 1R event with gnathostomes, but not the 2R event (Simakov et al. 2020); the gnathostome-specific set of miRNAs are those miRNA families found only in gnathostomes, but not in cyclostomes or any other metazoan taxon (see MirGeneDB for taxonomic assignments of all metazoan miRNA families as well as genes).

The miRNA phylogeny (supplementary fig. 1, Supplementary Material online) was constructed by first aligning the 254 miRNA precursor sequences present in the gnathostome LCA from each of the 32 descendant taxa by eye using both the positions of the RNaseIII cuts as well as the secondary structure of the pre-miRNA molecule as alignment guides. This data set, consisting of 16,146 nucleotide positions, was analyzed using the CAT-GTR+G (28,000 Cycles) (supplementary fig. 1, Supplementary Material online) and the GTR+G (2,800 cycles) models in Phylobayes (MPI—version 1.8) with similar results. Convergence was tested using tracecomp and bpcomp (Phylobayes). For the CAT-GTR analyses, we used a burnin of 10,000 cycles and a subsampling frequency of 10. All statistics reported by tracecomp had an effective sample size >1,000 and a relative difference <0.07 and the bpcomp maxdiff statistic was 0.02, indicating an excellent level of convergence.

To construct an amino acid data set of equal size to the miRNA data set, a jackknifing approach was taken. First, we took a set of protein alignments (Braasch et al. 2016) that represents a set of curated orthologous protein families designed for the analysis of vertebrate phylogeny. For each of these protein families, the *L.oculatus* sequence was extracted and the best BLAST (Altschul et al. 1990, 1997) hit was found using BlastP with a maximum e-value of 1e-10 in the proteome of each species present in this study but not that of Braasch et al. (2016). Each of these sequences was then blasted back against the *L. oculatus* proteome and the sequence was added to the orthologous protein family only if its best hit was the same protein that was used as the initial query. For the reverse BLAST, as above, the best hit was found using BlastP with an e-value cutoff of 1e-10. This resulted in 221 gene families for which all species had an ortholog present. These were aligned using mafft 7.429 (Katoh et al. 2002; Katoh and Standley 2013) with default settings and trimmed using TrimAl 1.4 (Capella-Gutierrez et al. 2009) with the -strict option implemented. These trimmed alignments were concatenated to form a superalignment of 80,040 amino acids. From this, five independent jackknife samples were taken using python scripts to randomly select 16,146 sites, which equals the length of the miRNA data set.

A Bayesian reconstruction of the phylogeny was performed for each data set using PhyloBayes MPI version 1.8 (Lartillot et al. 2013) for between 21,000 and 24,000 cycles under a model of CAT+GTR+4 discrete gamma categories. Two chains were run for each data set and, after 2,500 cycles were removed as burn-in, convergence was investigated using bpcomp and tracecomp (part of the PhyloBayes suite). Runs were deemed to have converged if all statistics reported by tracecomp had an effective sample size >50 and a relative difference <0.3. After convergence had been reached, a consensus tree was constructed using bpcomp, discarding 2,500 samples as burn-in, from all data sets.

The rate of miRNA nucleotide evolution was done by first aligning pre-miRNA sequences for 170 possible miRNA genes with at least one paralog on the α subgenome and one paralog on the β subgenome (supplementary file 5, Supplementary Material online). The resulting alignments for the subset of the 170 genes that could be analyzed for each of the 13 analyzed taxa were assessed using maximum likelihood in Paup 4.0a. The GTR model with an estimated rate matrix was used, with a gamma distribution set to 0.5, and the state frequencies empirically derived. A second analysis concatenated each of the 13 taxa into a single super-alignment (supplementary file 6, Supplementary Material online) and was analyzed in exactly the same manner (fig. 4*B*). 

## Supplementary Material

Supplementary data are available at *Molecular Biology and Evolution* online.

## Supplementary Material

msab344_Supplementary_Data
