# Supplementary material for: MicroRNAs as Indicators into the Causes and Consequences of Whole-Genome Duplication Events
Source: Mol Biol Evol. 2021 Dec 3;39(1):msab344. doi: 10.1093/molbev/msab344 (PMC8789304; doi:10.1093/molbev/msab344)
Supplement: msab344_Supplementary_Data [file msab344_supplementary_data.zip › SupplementaryFigs&Tables.pdf]

Supplemental Table 1. Paralogue retention in each of the four ancestral sub-genomes in the gnathostome LCA for genes that encode DNA repair proteins*.

DNA Repair#	1α	2α	1β	2β
Total	86	73	12	14
4X	0	0	0	0
3X	0	0	0	0
2X	6	8	2	4
1X	80	65	10	10

* These data are found in Supp. File 1.

# The difference in paralogue retention for genes that encode DNA repair proteins is significantly different between the α and β sub-genomes (chi-square = 95.6, df = 1, $P<0.0001$), but not between the 1 versus 2 sub-genomes (chi-square = 0.26, $P>0.6$).

Supplemental Table 2. Paralogue retention in each of the four ancestral sub-genomes in the gnathostome LCA for genes that encode transcription factors*.

TXFs#	1α	2α	1β	2β
Total	141	120	50	48
4X	6	6	6	6
3X	42	37	27	23
2X	70	63	14	13
1X	23	14	3	6

* These data are found in Supp. File 1.

# The difference in paralogue retention for genes that encode transcription factors is significantly different between the α and β sub-genomes (chi-square = 78.5, df =1, $P<0.0001$), but not between 1 versus 2 sub-genomes (chi-square = 0.65, $P>0.4$).

Supplemental Table 3. Median expression values (in reads per million) of unique miRNA mature sequences found on at least one α and one β sub-genome.

Taxon	1α	2α	1β	2β
Aca* (40#)	5,571	1,750	1,143	3,385
Ami (45)	247	242	23	171
Cmi (46)	13,833	12,339	3,955	5,513
Cpi (51)	11,183	4,917	1,185	5,018
Gga (28)	37,333	345,872	11,748	92,556
Hsa (37)	39,828	69,936	28,903	59,684
Loc (34)	10,038	9,602	4,224	5,169
Mdo (31)	5,576	22,675	364	940
Mun (38)	1,659	852	613	23
Oan (39)	2,179	31,547	1,580	1,983
Xtr (38)	4,761	24,886	2,523	1,218
Median†	5,574	10,971	1,383	2,684

* Taxon abbreviations: Aca, *Anolis carolinensis*; Ami, *Alligator mississippensis*; Cmi, *Callorhinchus milii*; Cpi, *Chrysemys picta*; Gga, *Gallus gallus*; Hsa, *Homo sapiens*; Loc, *Lepisosteus oculatus*; Mdo, *Monodelphis domestica*; Mun, *Microcaecilia unicolor*; Oan, *Ornithorhynchus anatinus*; Xtr, *Xenopus tropicalis.*

\# The number of miRNAs considered in each of the 11 taxa.

† The difference in median expression between the α and β sub-genome is significant (Chi-squared = 63577.9, df=1, P<0.0001).

Supplemental Table 4. Median branch lengths of pre-miRNA mature sequences found on at least one α and one β sub-genome in each of the indicated taxa.

Taxon	1α	2α	1β	2β
Aca* (115#)	0.105	0.164	0.222	0.196
Ami (126)	0.145	0.161	0.197	0.232
Cmi (128)	0.165	0.15	0.105	0.202
Cpi (129)	0.176	0.118	0.173	0.189
Gga (99)	0.187	0.145	0.128	0.184
Hsa (108)	0.192	0.138	0.204	0.284
Lch (149)	0.175	0.13	0.12	0.188
Loc (119)	0.188	0.108	0.156	0.256
Mdo (99)	0.158	0.062	0.248	0.318
Mun (118)	0.179	0.16	0.168	0.215
Oan (109)	0.164	0.143	0.204	0.218
Spt (116)	0.213	0.136	0.192	0.193
Xtr (97)	0.191	0.197	0.151	0.276
Median†	0.176	0.143	0.173	0.215

* Taxon abbreviations: Aca, *Anolis carolinensis*; Ami, *Alligator mississippensis*; Cmi, *Callorhinchus milii*; Cpi, *Chrysemys picta*; Gga, *Gallus gallus*; Hsa, *Homo sapiens*; Lch, *Latimeria chalumnae*; Loc, *Lepisosteus oculatus*; Mdo, *Monodelphis domestica*; Mun, *Microcaecilia unicolor*; Oan, *Ornithorhynchus anatinus*; Spt, *Sphenodon punctatus*; Xtr, *Xenopus tropicalis.*

\# The number of miRNAs considered in each of the 13 taxa.

† The difference in branch lengths between 2α and 2β – but not between 1α and 1β – is significant ($F(1,48) = 29.43$, $P<0.0001$) .

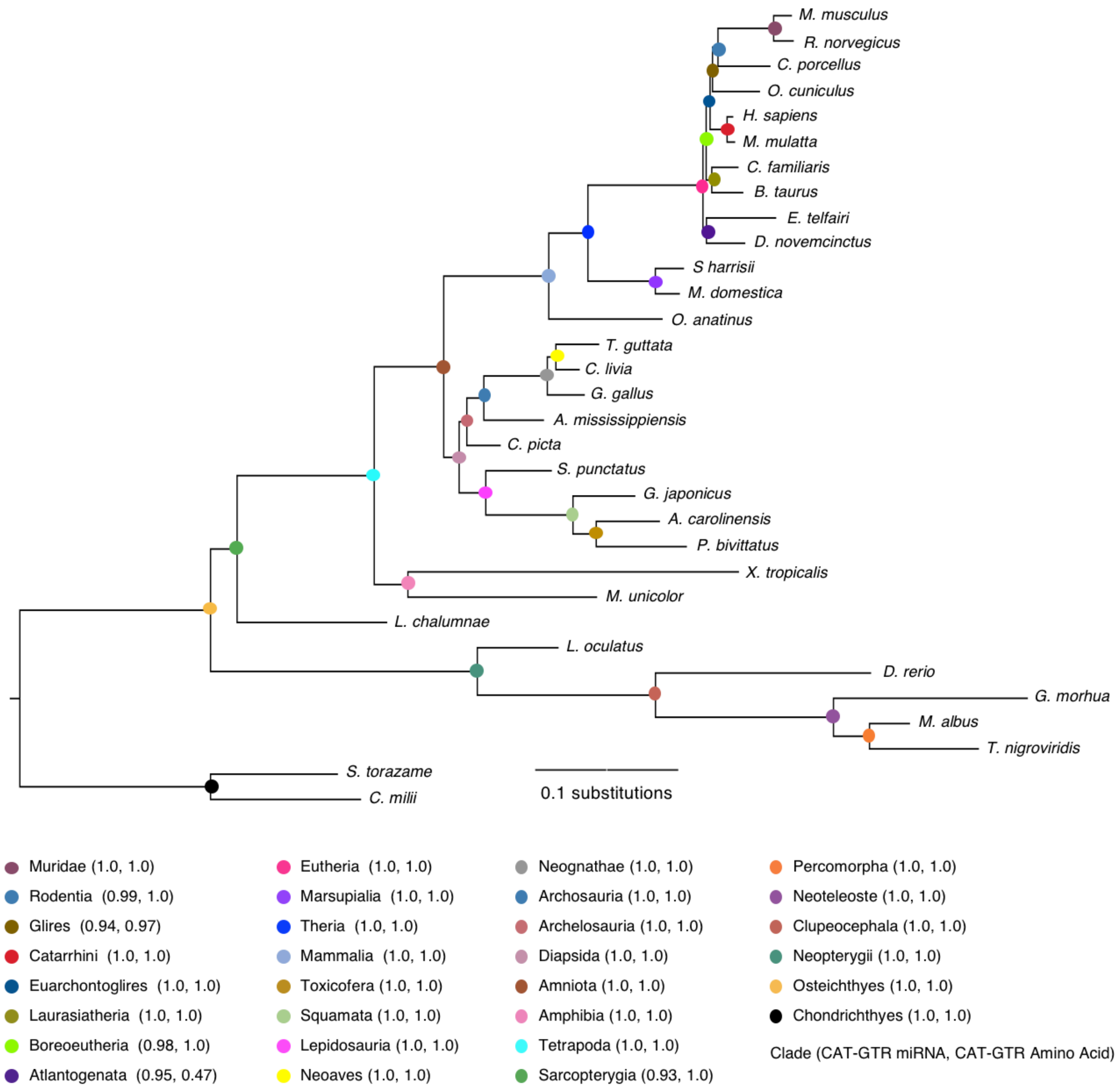

Supplemental Figure 1. Phylogenetic analysis of 254 pre-miRNA sequences reconstructed as present in the gnathostome LCA (see Mirgenedb.org) from 32 extant taxa (16,146 characters). Each node is color-coded, and the support values from two different analyses (the posterior probabilities [PP] from CAT-GTR, and GTR analyses, respectively, see materials and methods) are indicated as well. Outside of the Boreoeutheria, support values are high, consistent with the orthology assignments of the miRNA loci in MirGeneDB.org.

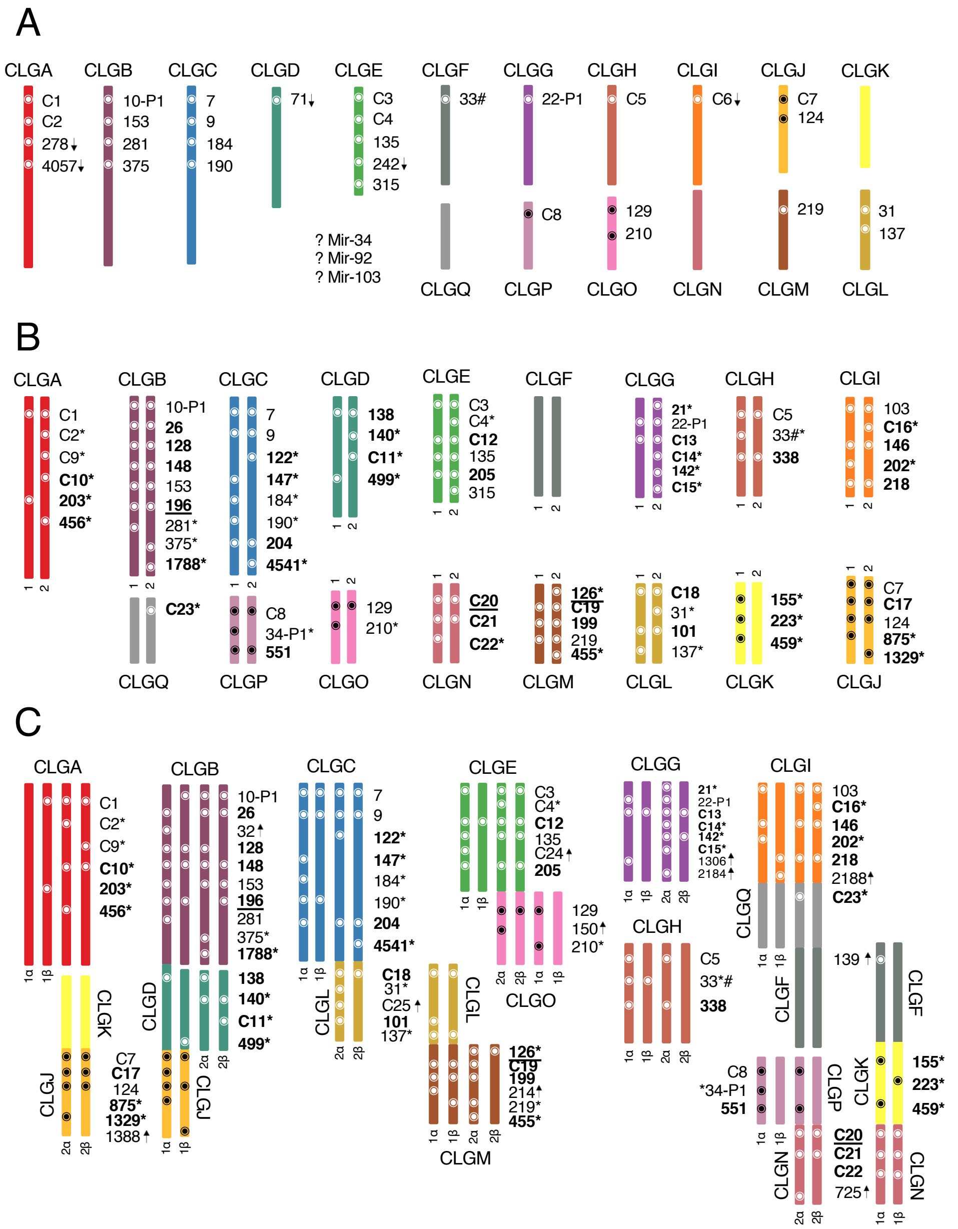

Supplemental Figure 2. Chromosomal locations of miRNA genes in the last common ancestor of the vertebrate pre-1R (A), vertebrates (B), and gnathostomes (C). A. The distribution of miRNA genes across each of the 17 ancestral chordate linkage groups (A-Q) as proposed by Simakov et al. (2020). Three ancient miRNA genes or clusters of genes cannot reliably be placed in the vertebrate pre-1R (although they can in the vertebrate LCA): Mir-34, Mir-93 and Mir-103 due to independent gene duplications found in *B. floridae* with resulting paralogues in different CLGs relative to the vertebrates. Four genes or clusters of genes are also lost and are indicated with downward arrows: *Mir-278*, *Mir-4057*, *Mir-71*, and *Mir-252*. Mir-33 appears to be on a different CLG in the pre-vertebrate 1R genome relative to where it is found in the vertebrate LCA genome (pound sign). **B.** The distribution of miRNA genes across each of the 34 ancestral vertebrate linkage groups following 1R as proposed by Simakov et al. (2020). Vertebrate-specific miRNA families are indicated in bold; those in bold underlines arose after the last common ancestor of chordates,

but before the last common ancestor of the Olfactores. The asterisks indicate vertebrate-specific genes present on only one of the two duplicate chromosomes (see Table 1 and Supp. File 3). **C.** The distribution of miRNA genes across each of the 45 ancestral gnathstome linkage groups following 2R as proposed by Simakov et al. (2020) with updates from Lamb (2021). MiRNA families that evolved after the second genome duplication event are indicated with the upward arrow. Cluster abbreviations are as follows: C1: Let-7-P1 + Mir-10-P2/P3; C2: 216-P1a/P1b + Mir-217; C3: Mir-29-P1/P2; C4: Mir-96-P1-P3; C5: Mir-193-P1/P2; C6: Mir-252-P1/P2; C7: Mir-1 + Mir-133; C8: Mir-8-P1-P3; C9: Mir-34-P2a/P2b; C10: Mir-192 + Mir-194; C11: Mir-208-P1/P2; C12: Let-7-P2a-P2c; C13: Mir-130-P1-P4; C14: Mir-132-P1/P2; C15: Mir-144 + Mir-451; C16: Mir-143 + Mir-145; C17: Mir-30-P1/P2; C18: Mir-23 + Mir-24 + Mir-27; C19: Mir-181-P1/P2; C20: Mir-15-P1/P2; C21: Mir-17-P1-P4 + Mir-19-P1/P2 + Mir-92-P1/P2; C22: Mir-221-P1/P2; C23: Mir-430-P1-P4; C24: Mir-191 + Mir-425; C25: Mir-34-P3a-d.

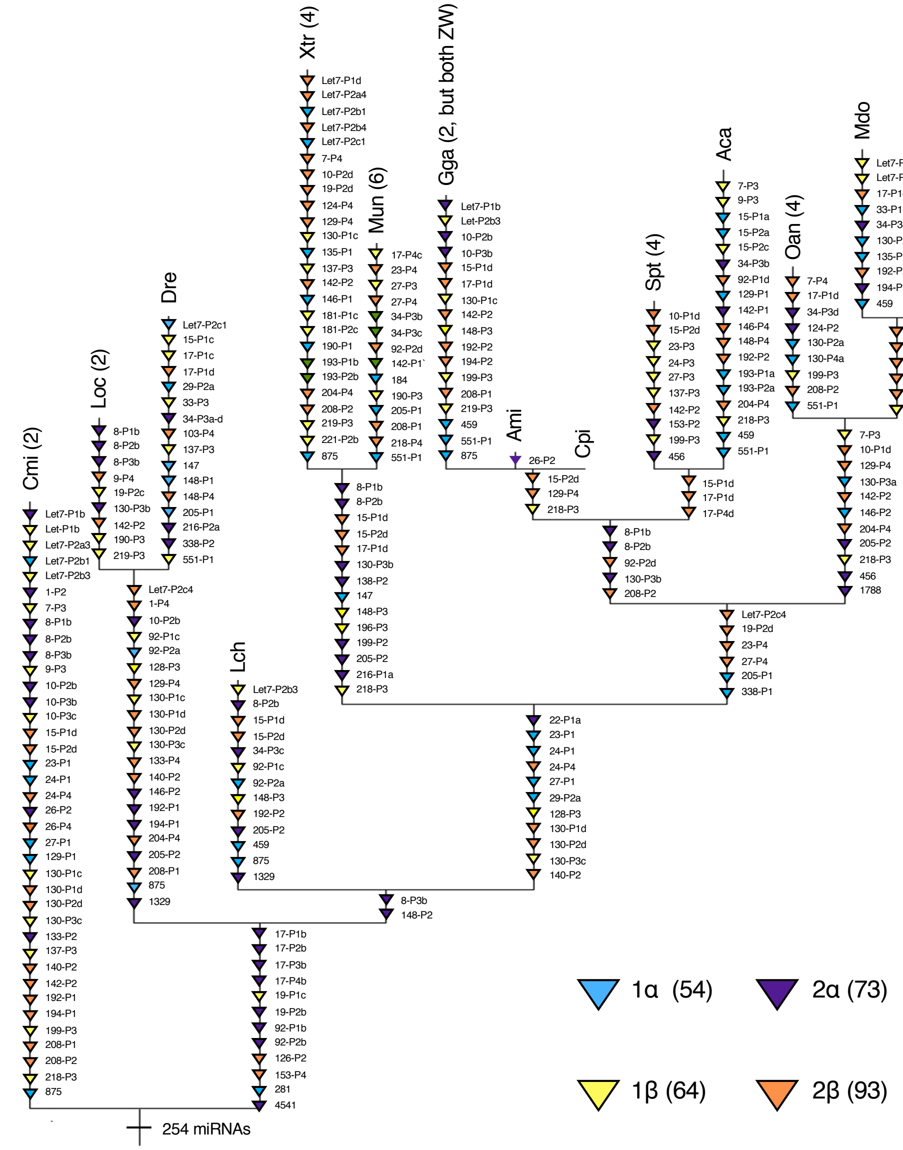

Supplemental Figure 3. The fate of the 254 miRNAs reconstructed in the gnathostome LCA and the sub-genome distribution of subsequent losses in 14 extant gnathostome taxa. Despite possessing only about half the miRNA loci (Table 2), miRNAs found on the β sub-genome are still lost at an enhanced rate relative to α sub-genome, a significant difference (χ^2 = 32.0, df = 3, P<0.0001) and one not found with protein-coding loci (Simakov et al. 2020). The key to the right tallies the total number of paralogue losses for each of the four sub-genomes across all 14 taxa (Supp. File 4). Taxon abbreviations are as follows: Aca, *Anolis carolinensi*; Ami, *Alligator mississippiensis*; Cmi, *Callorhinchus milii* (elephant shark); Cpi, *Chrysemys picta*; Dre, *Danio rerio* (zebrafish); Gga, *Gallus gallus*; Hsa, *Homo sapiens*; Lch, *Latimeria chalumnae*; Loc, *Lepisosteus oculatus* (spotted gar); Mdo, *Monodelphis domestica*; Mun, *Microcaecilia unicolor*; Oan, *Ornithorhynchus anatinus* (platypus); Spi, *Sphenodon punctatus*; Xtr, *Xenopus tropicalis*.

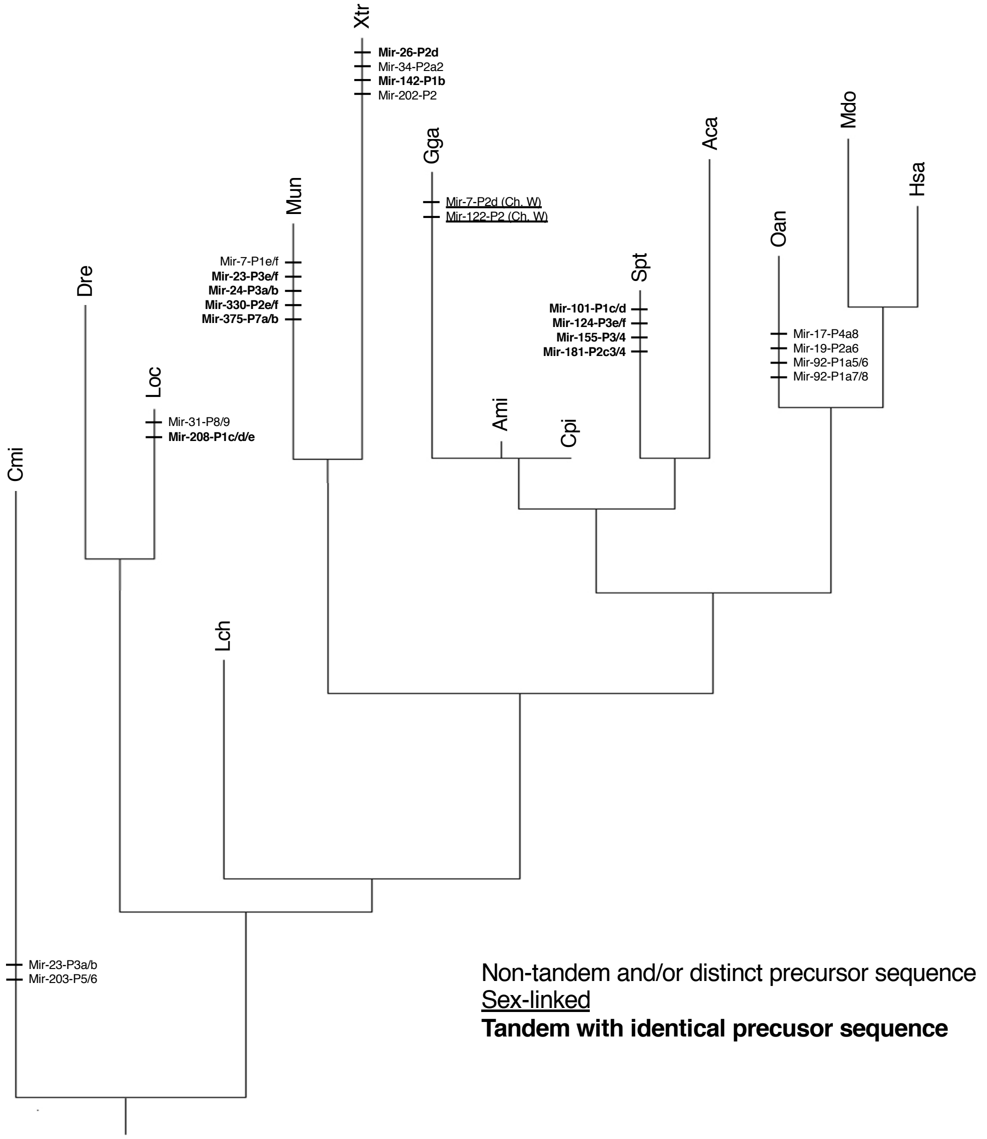

Supplemental Figure 4. Instances of miRNA duplications of the ancestral 254 miRNAs reconstructed in the gnathostome LCA following 2R. Indicated are the total number of reconstructed gene duplication events involving the original 254 miRNA genes following the gnathostome LCA as found in each of the 14 representative descendant taxa. No paralogues are shared between any two taxa, and nearly half (11 of 23) involve tandemly arranged copies of miRNAs with identical precursor sequences (bold), indicative that at least some of these might be the result of mis-assembly (Rhie et al. 2021). Others though appear to be bona fide (albeit recent) duplicates that are the result of usually tandem gene duplication, or through the individualization of sex chromosomes (underlined). Data from MirGeneDB v.2.1.

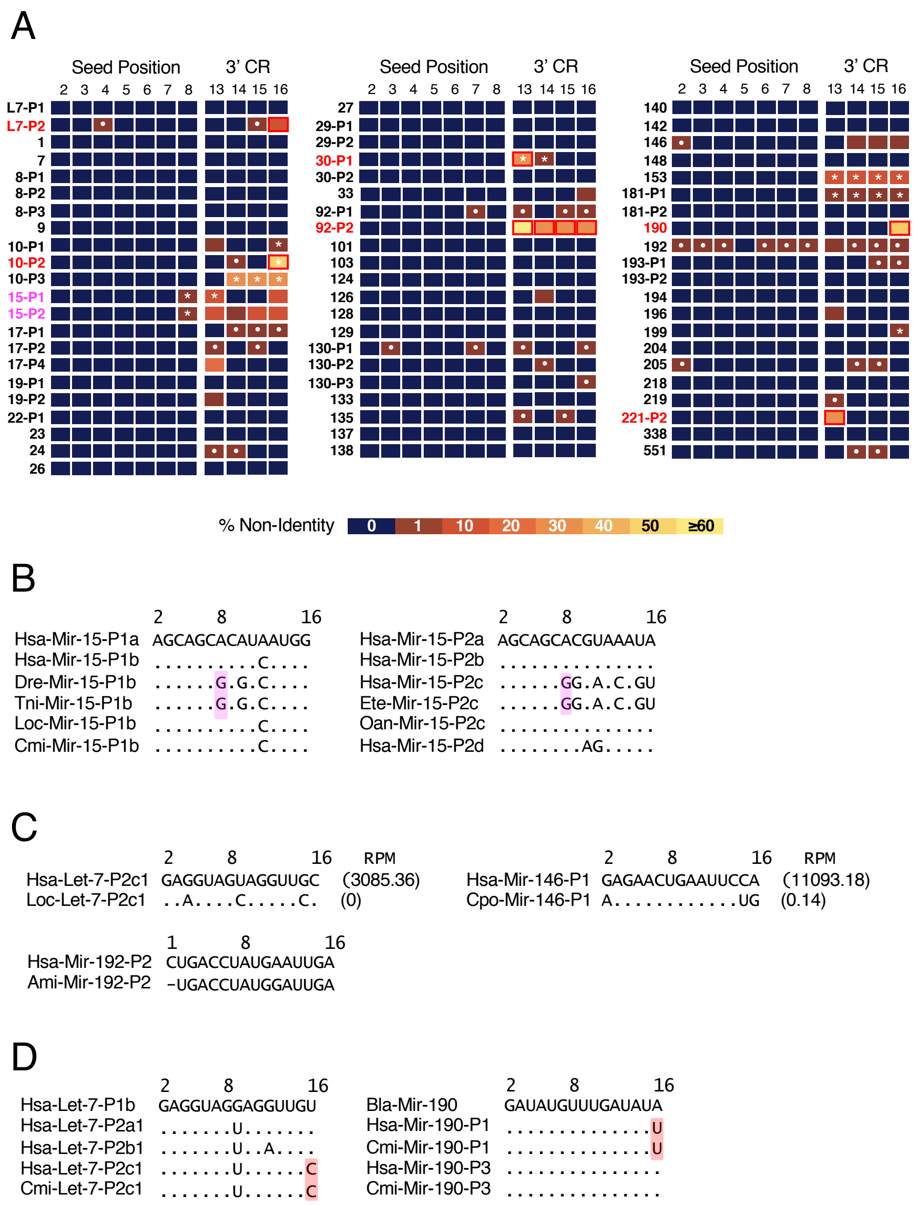

Supplemental Figure 5. **Changes to the seed (nucleotides 2-8) and the 3'-complementary regions (nucleotides 13-16) in miRNA paralogues generated from the gnathostome WGD events. A.** Sixty-four miRNA genes were present in the vertebrate lineage that had at least two paralogues in the gnathostome lineage resulting from either 1R and/or 2R. Changes to miRNA seed and 3'-complementary regions (CR) were assessed by these regions of the mature miRNA in relation to the consensus sequence for the entire family across the 33 gnathostome taxa present in MirGeneDB v.2.1, and the percentage difference indicated according to the heat map. No changes to the seed sequence – either through seed shifting (Wheeler et al. 2009) or mutations to the seed sequence itself – are the result of these WGD events. Instead, the few changes documented to the seed sequence are the result of mutations that occurred long after 2R. Two of these changes are shared mutations (# symbols) found in specific lineages (magenta), including position 8 in Mir-15-P1 in clupeocephalans or the same position in Mir-15-P2 in eutherian mammals (see panel **B**). Most

others though are specific changes that occurred in a single paralogue in a single taxon (white dots), including Let-7-P2c1 in the spotted gar and Mir146-P1 in guinea pig, which might be due to the pseudogenization of the locus (see panel **C**). The only potential instances of neo-functionalization are changes to the 3' CR regions (red) found in six miRNA paralogues generated by either 1R or 2R (see panel **D**). **B**. The only two examples of shared mutations to the seed sequence are position 8 in Mir-15-P1b in clupeocephalan fish and position 8 of Mir-15-P2c in eutherian mammals (magenta). Because these changes occurred long after 2R they are not examples of neo-functionalization as adaptations of WGD, but, at best, are examples of WGD exaptations. **C.** Some changes to the seed sequence are due to unique changes in a single species due to either due to seed shifting (e.g., Ami-Mir-192-P2) or possibly because of the pseudogenization of the miRNA locus itself (e.g., Loc-Let-7-P2c1 and Cpo-Mir-146-P1) as assessed by the dramatic difference in expression (measured by reads per million [RPM]) between these two potential pseudogenes as compared to their human counterparts. **D.** Potential instances of neo-functionalization are found in the 3'CR of six miRNA paralogues where changes characterize an entire paralogue sub-group relative to other members of the same family (red). For example, both Let-7-P2c1 and Mir-190-P1 have changes in position 16 of the mature miRNA sequence relative to other paralogue subgroups, and/or the single copy member found in invertebrates. These were seen though that resulted from the 2R events (red) including changes to position 16 in both (see panel **E**). Sequence position within the miRNA mature sequence are indicated with the numbers on top; RPM values for Mir-146 are indicated to the right of the sequence. Taxon abbreviations are as follows: Ami, *Alligator mississippiensis*; Bfl, *Branchiostoma floridae* (amphioxus); Cpo, *Cavia porcellus* (guinea pig); Cmi, *Callorhinchus milii* (elephant shark); Dre, *Danio rerio* (zebrafish); Ete, *Echinops telfairi* (tenrec); Hsa, *Homo sapiens*; Loc, *Lepisosteus oculatus* (spotted gar); Oan, *Ornithorhynchus anatinus* (platypus); Tni, *Tetraodon nigroviridis* (pufferfish).

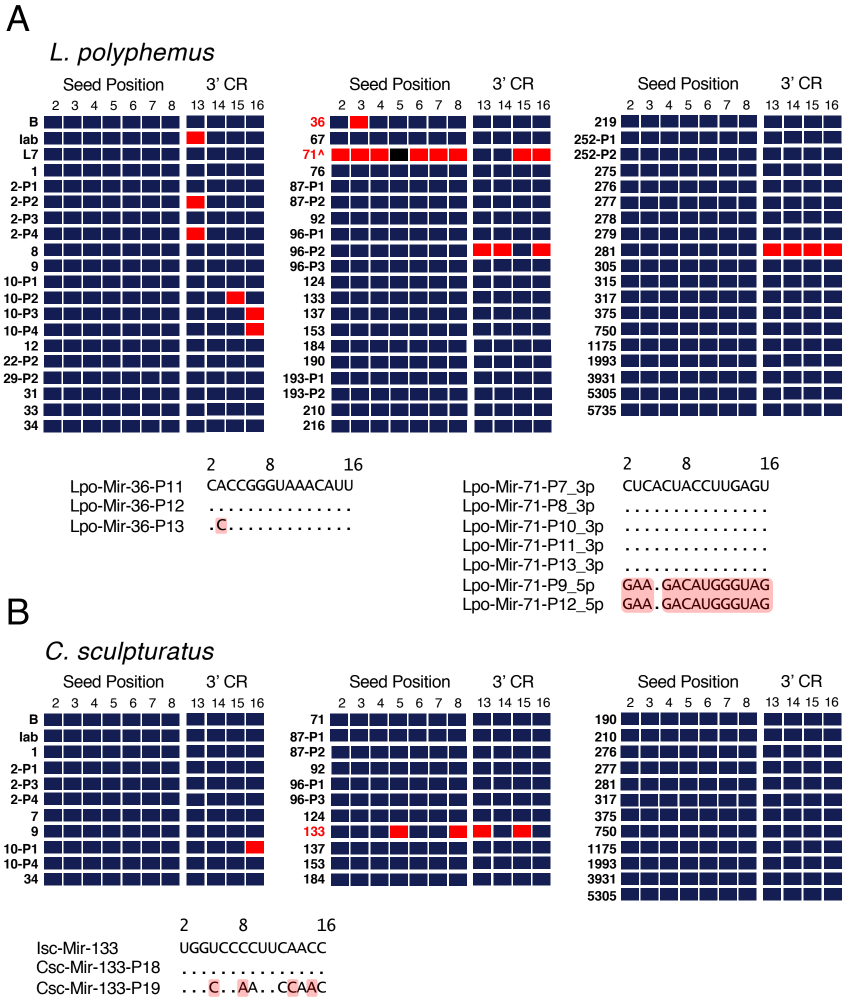

Supplemental Figure 6. **Changes to seed (nucleotides 2-8) and the 3'-complementary regions (nucleotides 13-16) in miRNA paralogues generated from WGDs in chelicerate arthropods. A.** Cataloguing all changes to the seed and 3'-CR regions in all miRNAs genes present in at least two copies within the horseshoe crab (*L. polyphemus*) genome (red) shows that, like the situation found in gnathostomes (Supp. Fig. 4), most miRNA paralogue sub-groups do not undergo any mutations. Only three instances of potential neo-functionalization are found within the miRNA repertoire of the horseshoe crab, including a change to position 3 in one of the three MIR-36 members, as well as arms switches (Marco et al. 2010; Griffiths-Jones et al. 2011) in two MIR-71 paralogues (^). All other changes are restricted to the 3' CR regions (red). Note though that because only a single species is represented here, these changes could have occurred long after the three WGD events in the horseshoe crab lineage and thus are not an adaptation, but again an exaptation. **B.** Similarly, cataloguing all changes to the seed and 3'-CR regions in all miRNAs present on two different scaffolds within the scorpion (*C. sculpturatus*) genome (red) shows that, again, most miRNA paralogue sub-groups do not undergo any mutations. This time only a single change to a miRNA mature seed region is seen, *Mir-133-P19*, which has mutation in position 5 of the seed, as well as positions 13 and 15 of the 3'CR. But again, without broader taxon sampling the timing of these changes relative to the single WGD in this lineage remains unknown. Taxon abbreviations: Csc, *Centruroides sculpturatus*; Isc, *Ixodes scapularis* (tick); Lpo, *Limulus polyphemus.*
